# Role of Novelty Seeking Personality Traits as Mediator of the Association between COMT and Onset Age of Drug Use in Chinese Heroin Dependent Patients

**DOI:** 10.1371/journal.pone.0022923

**Published:** 2011-08-17

**Authors:** Ting Li, Shunying Yu, Jiang Du, Hanhui Chen, Haifeng Jiang, Ke Xu, Yingmei Fu, Dongxiang Wang, Min Zhao

**Affiliations:** 1 Shanghai Mental Health Center, Shanghai Jiao Tong University School of Medicine, Shanghai, China; 2 Department of Psychiatry, School of Medicine, Yale University, New Haven, Connecticut, United States of America; The Scripps Research Institute, United States of America

## Abstract

**Background:**

Personality traits such as novelty seeking (NS) are associated with substance dependence but the mechanism underlying this association remains uncertain. Previous studies have focused on the role of the dopamine pathway.

**Objective:**

Examine the relationships between allelic variants of the catechol-O- methlytransferase (COMT) gene, NS personality traits, and age of onset of drug use in heroin-dependent subjects in China.

**Methods:**

The 478 heroin dependent subjects from four drug rehabilitation centers in Shanghai who were genotyped for eight tagging single nucleotide polymorphisms (SNP) on the COMT gene completed the NS subscale from the Temperament and Character Inventory. Multivaritate analyses were used to assess the potential mediating role of NS personality traits in the association between COMT gene variants and the age of onset of heroin use.

**Principal Findings and Conclusions:**

In the univariate analysis the COMT rs737866 gene variants were independently associated with both NS and age of onset of drug use: those with the TT genotype had higher NS subscale scores and an earlier onset age of heroin use than individuals with CT or CC genotypes. In the multivariate analysis the inclusion of the NS subscore variable weakened the relationship between the COMT rs737866 TT genotype and an earlier age of onset of drug use. Our findings that COMT is associated with both NS personality traits and with the age of onset of heroin use helps to clarify the complex relationship between genetic and psychological factors in the development of substance abuse.

## Introduction

Drug dependence is a chronic disease characterized by a compulsion to seek and use drugs that typically leads to severe negative consequences. Among the various types of drug dependence, heroin dependence causes the most serious health, social and legal consequences [1], [2]. The 2010 *World Drug Report* (UNODC) estimates 12.8–21.8 million opiate users in the world, more than half of whom are from Asia. According to the *2010 Annual Report on Drug Control in China*, there were 1.33 million registered drug users in China at the end of 2009, 70% of whom were opiates users. Notably, there are common vulnerability factors for individual to develop heroin dependence, such as early onset of drug use. Previous studies demonstrated that early onset of drug use is associated with severe consequences of substance use, e.g. these who is easy to develop into poly-substance users and difficult to quitr substance. Several studies have shown that drug dependence is the end result of an interaction between genetic and environmental risk factors [3], [4]. Based on twin studies and family studies genetic factors account for 50%–70% of the risk for drug dependence [5]. The dopamine pathway plays a crucial role in the reinforcement mechanism that leads to drug dependence [6] so genes involved in the dopamine pathway are excellent candidate genes to study the genetic precursors of drug use. Several polymorphisms of the dopamine receptor genes (DRD2, DRD4) and the dopamine transporter gene (DAT1) have been linked with addictive behavior [7], [8], [9]. Polymorphisms on the DRD2, DRD4, and DAT genes regulate dopamine system function by reducing dopamine receptor density or enhancing dopamine clearance, so they may increase the risk of addictive behavior via their influence on reward mechanisms [7], [10], [11].

Catechol-O-methyltransferase (COMT) catalyzes the breakdown of catechol neurotransmitters and, thus, plays an essential role in dopamine inactivation. The rs4860 (val158met) functional SNP on COMT, which results in a three- to four-fold increase in enzyme activity [12], has been extensively studied in psychiatric disorders including drug dependence. But the findings about its relationship with drug dependence have been inconsistent: a case-control study among Caucasian subjects found an association between the Val allele and increased risk for polysubstance abuse [13] and a family study in Israeli ethnic groups reported an association with heroin dependence [14], but a study in China did not identify an association with opiate dependence [15].

Other studies have shown that a variety of factors—gender, personality traits, childhood psychopathology—are associated with the age of starting drug use [16], [17], [18]. How an individual responds to events is determined by personality, which in some extent reflect psychobiological predispositions [19], so it is not surprising that different personality traits have been associated with drug use [20], [21], [22], [23]. Novelty seeking (NS), impulsivity, and harm avoidance (HA) have been identified as personality traits that are related both to the proneness to drug dependence and to the onset age of drug use [20], [21]; Wills and colleagues found that high NS and low HA personality traits increase the risk of using drugs [22], [23]. Our previous work also found that heroin dependent patients had high NS compared with controls [24].

According to Cloninger's model of personality, NS involves exploratory behaviors and activation in response to novel stimuli that is reinforced by specific constellations of genetically-determined neurotransmitters, particularly those in the dopamine system [25], [26]. Different studies have documented a robust relationship between NS and dopamine system genes in drug abuse: women with the COMT Met/Met genotype had higher NS than those with the Val/Val or Val/Met genotypes [27]; methamphetamine dependent patients with the COMT Met and DRD2-TaqI A1 alleles have higher NS scores [26], [28]; and NS personality traits mediate the association between the dopamine D4 receptor gene exon III polymorphism and drinking and smoking behaviors in male adolescents [29], [30], [31].

The relationship of the COMT gene with NS personality traits in heroin dependent subjects has not, as yet, been investigated. To help clarify this relationship the current study assesses the association of eight tagging SNPs on COMT gene with NS in a large sample of heroin dependent subjects from China. Given that early initiation of drug use predicts the development of dependence [32], [33] and our previous finding about the relationship of NS personality traits with heroin dependence [24], we hypothesize that NS personality traits may affect an individual's predisposition to heroin dependence via their effect on reducing the onset age of drug use. The study aims (1) to assess whether the COMT gene SNP variants are associated with NS (as measured by the Temperament and Character Inventory) and onset age of drug use in heroin dependent patients; and, if an association is found, (2) to examine whether NS personality traits mediate the association between COMT gene variants and onset age of drug use in heroin dependent patients.

## Methods and Materials

### Ethics Statement

The research protocol was approved by the Ethic Committee of the Shanghai Mental Health Center and each subject signed the informed consent form approved by IRB at the Shanghai Mental Health Center.

### Subjects

A total of 478 heroin dependent patients from four drug rehabilitation centers in Shanghai were recruited. They included 234 men and 244 women and had a mean (SD) age of 33.8 (7.8) years. All subjects were interviewed with SCID-I by trained psychiatrists and met the criteria for heroin dependence according to the fourth edition of the Diagnostic and Statistical Manual of Mental Disorders (DSM-IV). Subjects with other axis I psychiatric diagnoses were excluded from the study. Urine and blood samples were obtained and screened for illicit drugs and genotype. Eligible subjects completed a self-completion form developed for the study that included basic demographic information (age, years of education, marital status, etc.) and information on the history of drug use (onset age of drug use, frequency and amount of current daily use, times of previous drug treatment, etc.).

### Measures

The NS personality trait was assessed by the corresponding subscale of the Temperament and Character Inventory (TCI). The TCI was developed by Cloninger and colleagues that has been translated and validated in Chinese [34], [35]; the Chronbach alpha of the Chinese version are 0.56–0.81; and the Cronbach alpha of NSscale is 0.69 [35]. TCI includes seven subscales including Novelty Seeking (NS), Harm Avoidance (HA), Reward Dependence (RD), Persistence (PS), Self-Directedness (SD), Cooperativeness (CO), and Self-Transcendence (ST). The NS subscale includes 20 items with scores from 1 to 4, thus the total score ranges from 20 to 80; higher scores representing more prominent NS personality traits.

### SNP selection and genotyping for COMT gene

**Figure 1:**
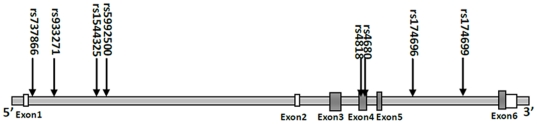
Human COMT gene structure and location of eight tagging SNPs. [The gray boxes and the white boxes indicate the exons of the COMT gene. The arrows indicate the location of each tagging SNPs.]

Genomic DNA for each subject was extracted from venous blood using a modified phenol/chloroform method. Tagging SNPs for COMT were selected from the region of chr22:18309309..18336528 in the HapMap database for Han Chinese population (http://hapmap.ncbi.nlm.nih.gov). In this study, tagging SNP was defined as having an r^2^ threshold of greater than 0.6. Eight tagging SNPs with minor allele frequency greater than 0.1 (rs4818,rs4680,rs174696,rs174699,rs737866,rs933271,rs1544325, rs5992500) were indentified (Fig. 1). Genotyping for the eight SNPs were performed by using the TaqMan Genotyping Assay (Applied Biosystems, Foster City, California) on the ABI Prism 7900 sequence detection system for all subjects. For quality control, 5% random DNA samples were genotyped twice for each SNP to calculate genotyping error. The genotyping accuracy was 100%.

### Statistical analyses

ANCOVA and independent t- tests were used to compare the mean NS subscale scores and the mean onset age of heroin use among groups defined according to different COMT gene SNPs genotypes. Estimates of pair-wise LD based on the r-squared statistic were obtained using Haploview software, version 3.32 [36]. haplotype-based quantitative trait association analysis was performed for the haplotype linear regression analysis using PLINK version 1.06 software (www.pngu.mgh.harvard.edu/~purcell/plink) [37], We also performed permutation test (calculated by PLINK) based on 10 000 permutations for multiple test adjustment. Significance was considered at P-value 0.05.

The mediation analysis model [38] was used to test whether NS mediates the effect of COMT genotypes on heroin dependence. Briefly, a mediation hypothesis can be accepted if three conditions are satisfied based on the results from three regression analyses: (1) the independent variable (COMT genotype) must affect the mediator (NS); (2) the independent variable must affect the dependent variable (onset age of drug use); and (3) when the independent variable (COMT genotype), dependent variable (onset age of drug use) and mediator variable (NS) are all in the regression model, the effect of the independent variable on the dependent variable must be less than when the mediator variable is not in the model (model 2). Perfect mediation occurs if the independent variable has no effect on the dependent variable in the third regression model (i.e., the mediator fully controls the relationship between the independent and dependent variables). Mediation was tested by Pearson correlations, liner regression and logistic regression analyses.

## Results

### Demographic and drug use data

Demographic and drug use history of the subjects is summarized in Table 1.

**Table 1:** Demographic characteristics and drug use history among 478 heroin dependent subjects from Shanghai China.

Mean(SD)years of education(years)	10.3(2.1)
Marriage status	
Married, n(%)	137(28.7)
Divorced or separated, n(%)	96(20.0)
Unmarried, n(%)	245(51.3)
Mean onset age of drug use(years)	22.9(51.3)
Mean duration of drug use (years)	8.92(6.4)
Mean daily intake of heroin in grams	0.92(0.5)
Mean daily frequency of drug use	3.9(1.7)
Mean number of previous drug treatments(times)	6.6(7.3)

### Associations of COMT SNPs with NS and age of onset for drug use in the heroin dependence subjects

As reported in previous studies [39], [40] we found a significantly higher mean (SD) NS subscale scores in males than in females (55.2 [6.7] vs. 57.1[7.5], df = 476; t = −3.04, p<0.019) so ANCOVA was used to compare the mean NS subscale scores and mean onset age of heroin use between participants with different COMT genotypes after adjusting for gender (Table 2). Three COMT gene SNPs variants (rs4818, rs174696 and rs737866) were significantly associated with NS subscale scores in these heroin dependent subjects. No association was found between rs4680, rs174699, rs933271, rs599250 and NS personality traits in heroin dependent subjects.

**Table 2:** Comparison of NS subscale score and onset age of heroin use in heroin dependent subjects with different COMT SNP genotypes.

SNP	Genotype Mean(SD)			P	Permutation P^a^
rs737866	T/T(n = 221)	C/T(n = 218)	C/C(n = 39)		
NS	57.2 (6.8)	55.5 (7.5)	54.5 (7.4)	0.004	0.005
Onset age	21.9 (5.5)	24.0(6.4)	22.3 (6.3)	0.014	0.019
rs933271	T/T(n = 182)	T/C(n = 214)	C/C(n = 82)		
NS	55.6 (7.9)	56.5 (6.5)	56.9 (7.3)	0.109	0.131
Onset age	23.0(6.2)	23.1(6.3)	21.9(5.4)	0.291	0.311
rs1544325	G/G(n = 224)	A/G(n = 191)	A/A(n = 43)		
NS	56.1 (6.9)	56.2 (7.4)	56.7 (7.6)	0.664	0.857
Onset age	23.0 (6.2)	22.9 (6.0)	22.1 (6.0)	0.427	0.429
rs599250	C/C(n = 409)	C/T(n = 69)	T/T(n = 0)		
NS	56.3 (7.2)	55.8 (7.0)	—	0.606	0.563
Onset age	23.2(6.2)	21.3(5.20)	—	0.018	0.022
rs4818	C/C(n = 204)	C/G(n = 213)	G/G(n = 61)		
NS	57.3 (6.9)	55.3 (7.4)	55.4(6.8)	0.01	0.012
Onset age	22.3 (5.9)	23.5 (6.4)	22.4(5.4)	0.336	0.458
rs4680	G/G(n = 244)	A/G(n = 207)	A/A(n = 27)		
NS	55.8 (7.1)	56.7 (7.3)	56.1 (7.5)	0.287	0.298
Onset age	22.9 (5.9)	22.8(6.4)	22.6(6.1)	0.776	0.857
rs174696	C/C(n = 131)	C/T(n = 239)	T/T(n = 108)		
NS	57.7 (7.1)	55.7 (7.2)	55.5 (7.2)	0.015	0.017
Onset age	22.7 (5.9)	23.3 (6.2)	22.3 (6.0)	0.727	1
rs174699	C/C(n = 165)	T/C(n = 222)	T/T(n = 91)		
NS	56.5 (7.2)	56.1(7.0)	55.9 (7.7)	0.497	0.7
Onset age	23.8 (6.5)	22.4(6.1)	22.5(5.1)	0.047	0.085

a Correction for multiple testing was performed using permutation test based on 100 000 permutations.

Previous studies reported that gender differences are present in different age groups of substance abusers [41] so we used ANCOVA to adjust for gender (as a covariate) when comparing the mean onset age of heroin use across the different COMT genotypes. Table 2 shows that three COMT gene SNPs variants (rs4818, rs737866, rs599250 variants) had significant effects on onset age of heroin use. No association was found between onset age of heroin use and the COMT rs4680, rs174696, rs933271 or rs154432 genes.

### Associations of COMT haplotypes with NS and age of onset for drug use in the heroin dependence subjects

**Figure 2:**
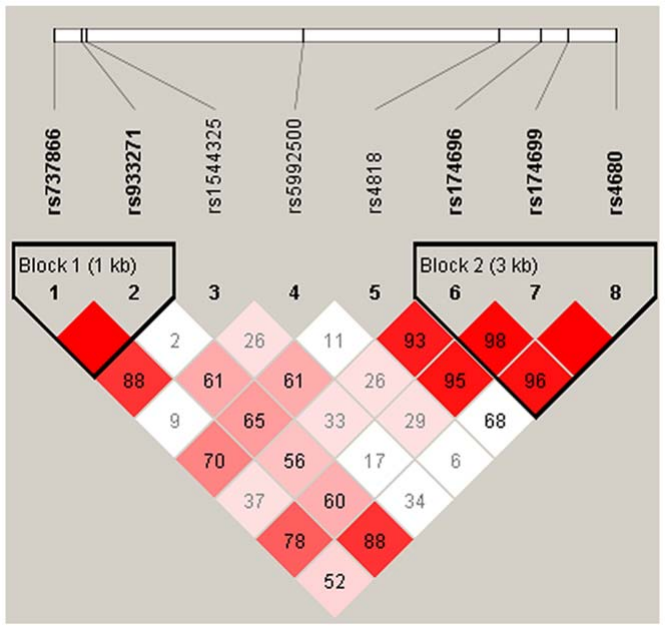
Linkage disequilibrium (LD) plot of the tagging eight SNPs.

To perform haplotype-based association analyses, we examined LD structures within all genotype data and identified for two haplotype-blocks (Figure 2). Block 1 was 1 kb long, consisted of two SNPs (rs737866 and rs933271), and included three common haplotypes TC, CT, and TT. Block 2 was 3 kb long, consisted of three SNPs (rs174696, rs174699 and rs4680), and included six haplotypes with frequencies greater than 0.05 (Table 3). Haplotype linear regression analysis showed that the haplotype CT in block 1 was significantly associated NS and onset age for drug use, haplotype TTG and CTG in block2 was significantly associated NS, and haplotype TTG in block2 was slightly associated onset age for drug use.

**Table 3:** Haplotype Analysis of COMT SNPs with NS subscale score and onset age of heroin use in heroin dependent subjects.

Haplotype		Novelty Seeking subscale score				Onset age of heroin use			
	F	Beta	Stat	P	Permutation P^a^	Beta	Stat	P	Permutation P^a^
**rs737866-rs933271**									
**TC**	0.388	0.854	3.36	0.067	0.067	−0.56	2.01	0.157	0.162
**CT**	0.302	−1.38	7	0.006	0.008	0.926	4.39	0.034	0.037
**TT**	0.303	0.387	0.597	0.44	0.44	−0.359	0.715	0.398	0.407
**rs174696-rs174699-rs4680**									
**TTA**	0.101	0.327	0.137	0.711	0.717	−0.384	0.266	0.606	0.603
**CTA**	0.132	1.28	2.58	0.11	0.109	0.398	0.349	0.555	0.562
**TCG**	0.121	−0.899	1.41	0.236	0.247	−1.12	3.04	0.082	0.086
**CCG**	0.262	0.192	0.111	0.74	0.733	−0.38	0.606	0.437	0.438
**TTG**	0.229	−1.38	5.52	0.02	0.019	0.988	3.94	0.048	0.05
**CTG**	0.115	2.04	6.19	0.013	0.014	0.467	0.449	0.503	0.501

a Correction for multiple testing was performed using permutation test based on 10 000 permutations.

### NSas a potential mediator of the association between COMT and onset age of heroin use

Pearson correlation analyses showed that the NS subscale scores were correlated with onset age of drug use (r = −0.191, p<0.001). Individuals with higher NS score had earlier onset age of drug age. In addition, the results showed that COMT genotype at rs737866 variants were associated with both NS subscale scores and the onset age of drug use. Given the existence of these associations, we proceeded to use linear and logistic regression models to assess whether or not the NS subscale scores met the criteria of being a ‘mediator’ of the association between COMT gene variants and onset age of heroin use. In order to examine factors affect an earlier onset drug use, based on our results (the median age of onset drug use is 22 years old) and other studies [42], 22 years old was used as cut-off to define earlier drug use, the onset age of heroin use was re-coded as a dichotomous variable (1≤22 years old, 2>22 years old). The COMT rs737866 SNP genotype was re-coded as dichotomous variable (1 = “TT”, 2 = “”CT” or “CC”). Table 4 presents the results of the liner regression and the two binary logistic regression models and shows that the three conditions for mediation were met. First, COMT genotype was a significant related to the NS subscale scores (r = −0.125, p = 0.006), individuals with TT genotype would have higher NS score than who with CT or CC genotype; second, the COMT genotype was significantly related to the onset age of drug use (odds ratio [OR] = 0.63, 95% confidence interval [CI]:0.44–0.91; p = 0.013); and third, the association between the COMT genotype and onset age of drug use became weaker (p value increased from 0.013 to 0.038) when NS was taken into account in the regression model (OR = 0.67, 95% CI: 0.46–0.98; p = 0.038). Finally, 3.5% of the overall variance in age of onset was due to the combined effect of the COMT gene variants and NS personality traits in the regression model.

**Table 4:** Logistic regression analysis testing the potential role of NS as a mediator between COMT rs737866 and the onset age of drug abuse*.

	Predictor	Beta	t/OR	R^2^	P
***Mediation condition 1***					
(dependent: NS subscale score)	rs737866	0.125	2.755	0.016	0.006
***Mediation condition 2***					
dependent: early or later onset of heroin use)	rs737866	−0.465	0.628	0.013	0.013
***Mediation condition 3***					
dependent: early or later onset of heroin use)	rs737866 AND	−0.396	0.673	0.035	0.038
	NS score		0.957		0.001

## Discussion

We assessed the associations of COMT gene variants with NS personality trait in Chinese heroin dependent subjects and found that the TT genotype of rs737866 was associated with higher NS and earlier onset age of heroin use. Additional regression analyses suggested that the association between COMT genotype onset age of drug use was partially mediated by the NS personality trait, in addition the direct effect of the genotype on the onset age of drug use.

Previous studies of the relationship between COMT gene and NS personality traits have had mixed results [27], [43], [44], [45]. Most of these previous studies have focused on the rs4680 functional polymorphism of COMT gene [12]. Golimbet and colleagues found that the Met allele of COMT gene (which decreases the enzyme activity and, thus, leads to higher dopamine levels), was associated with stronger NS personality traits in women [27]; this finding was replicated by Tsai and colleagues in young Chinese females [43]. However, the association of the COMT rs4680 gene with NS was not confirmed in another Caucasian population [45] or in a Japanese population [44]. The results did not showed rs4680 alone associated with NS, but the haplotype analysis indicates rs4680 together with other two adjacent SNPs (rs174696, rs174699) were significantly associated with NS, it suggested that rs4680 may not play a major effect but have slight contribution on NS in Chinese heroin addiction population between the rs737866 SNP and NS. Many potential factors could generate these inconsistent results across studies: heterogeneity of the phenotype; heterogeneity of the genetic background of subjects; insufficient sample sizes, and so forth.

The rs737866 SNP is located at the first intronic region of COMT gene and the promoter region of thioredoxin reductase 2 gene (TXNRD2)—a member of a family of pyridine nucleotide-disulfide oxidoreductases and a key enzyme in regulating the intracellular redox environment. COMT gene is located on the forward strand and TXNRD2 is on the reverse strand; there is a 8 kb overlap of the 5′ end of TXNRD2 and the un-coding region of MB-COMT. Some reports find that over-expression of the chromosome 22q11.2 segment (including TXNRD2, COMT and ARVCF) affects incentive learning and working memory in mice during development [46]. Even though rs737866 is an intronic variant, its possible affect the transcription of COMT or TXNRD2 gene, and it could be in linkage disequilibrium with nearby functional SNP.

Our results confirmed our first hypotheses about the relationship of COMT gene variants with NS personality traits and with the onset age of drug use. These findings support the proposition of several authors that the dopamine genes that affect heroin use operate through an association with some aspect of personality (like NS) that is also modulated by dopamine transmission [27]. Dopamine pathways that arise in the ventral tegmental area and project to the nucleus accumbens and the frontal cortex are fundamental components of reward processes [47]. The COMT enzyme accounts for the degradation of dopamine and plays a key role in prefrontal cortical functioning, so it probably affects the cognitive processes involved in substance dependence. Decreased prefrontal dopaminergic activity and low prefrontal cortical control associated with specific COMT genotypes could increase impulsivity, and the resulting NS behaviors could result in a compensatory increase in dopaminergic neurotransmission in the nucleus accumbens [48].

Individuals with high NS would have higher levels of exploratory activity and, thus, be more likely to start drugs earlier and to use them more extensively. Similarly, individuals with COMT gene variants that increase the enzymatic activity may experience a longer lasting and more effective dopamine release in the brain that lowers the threshold of pleasure-seeking behavior and increases the intensity of the activation of the reward network; this would also be likely decrease the age of onset and increase the severity of drug abuse. Our results also indicated NS had a strong direct effect to earlier age of onset, which supported the findings of previous studies that an association between NS and age of onset of drug use [24], [49], [50]. Higher NS subscale scores is linked with higher impulsiveness, exploratory excitability, extravagance and some personality disorders (e.g., antisocial, borderline) [51], [52]. NS scores tend to be stable over time so it is likely that these traits precede the onset of heroin use.

Since COMT gene variants (rs737866) and NS subscale scores were both associated with onset age of heroin use, we examined our second hypotheses and it was also supported. The association between COMT genotype and onset age of drug use became weaker (p value increased from 0.013 to 0.038) when the intensity of NS personality traits was taken into account. But the odds ratio for the effect of the genotype on the age of onset of drug use only increased marginally (from 0.63 to 0.67) after adjustment for the NS variable, so the results didn't meet the perfect mediation hypothesis; NS personality traits only partially mediate the association between COMT gene and heroin use. These findings are in line with previous studies about the relationship among dopamine system genes, NS personality traits, and alcohol or tobacco use. For example, studies have showed that the D4 dopamine receptor (DRD4) 7 repeat (7R) VNTR is associated with higher levels of NS and higher risk for smoking and alcohol intake in adolescence [29], [30], [31]. Another study found that the dopamine transporter (DAT) gene is associated with early, heavy or regular tobacco and alcohol use in young adulthood [53].

We suggest the following mechanism as a possible explanation for the complex relationship between the COMT gene (rs737866), NS personality traits, and heroin use: the COMT gene rs737866 variants located at promoter areas affect the COMT gene function and heroin use directly or through the mediating effect of NS personality traits. Our results indicate that the COMT rs737866 TT genotype is related to higher NS scores and that individuals with higher NS scores have an earlier onset age of drug use-which would increase the likelihood and severity of subsequent heroin dependence.

There are several limitations that need to be considered when interpreting our results. A previous study suggested possible population admixture in Chinese samples [54], it also a potential confounder that we were unable to address in our study. For lacking non-addicted controls, we were unable to determine whether or not the association between COMT variants and NS personality trait were specific to the substance abuse population or, alternatively, also true in the general population. Thirdly, in this study we did not administer the full TCI; for some research stressed NS would more likely to only represent the personality of pursuit new stimulant and impulsive. Several studies found an association of COMT with other subscales, such as harm avoidance.

Despite these limitations the study provides some new insights into the complex mechanisms operating at the interface between biology, personality and behavior. The biological effect of the COMT gene rs737866 variants highlighted in our results need to be assessed in more detail and their role in the addiction pathway must be clarified. It should, however, be emphasized that despite being independently associated with age of onset of heroin use, the combined effect of the COMT gene variants and NS personality traits only accounted for 3.5% of the overall variance in age of onset, so there are many other factors that need to be incorporated into our models before we can understand the onset and course of these disabling conditions.
